# Synthesis of a Keggin-type polyoxoselenidotungstate *via* site-selective oxygen-to-selenium substitution†

**DOI:** 10.1039/d5sc03340c

**Published:** 2025-06-16

**Authors:** Kentaro Yonesato, Yota Watanabe, Magda Pascual-Borràs, R. John Errington, Kazuya Yamaguchi, Kosuke Suzuki

**Affiliations:** a Department of Applied Chemistry, School of Engineering, The University of Tokyo 7-3-1 Hongo Bunkyo-ku Tokyo 113-8656 Japan ksuzuki@appchem.t.u-tokyo.ac.jp k-yonesato@g.ecc.u-tokyo.ac.jp; b NUPOM Lab, Chemistry, School of Natural & Environmental Sciences, Newcastle University NE1 7RU Newcastle Upon Tyne UK; c Department of Advanced Materials Science, Graduate School of Frontier Sciences, The University of Tokyo 5-1-5 Kashiwanoha Kashiwa Chiba 277-8561 Japan

## Abstract

Selenium, a group 16 (chalcogen) element, can endow metal oxides with unique properties when replacing oxygen atoms from specific sites. Polyoxometalates (POMs), a class of anionic metal oxide clusters, exhibit structure-dependent properties and applications. Despite the potential of chalcogen substitution, the replacement of oxygen atoms in POMs with chalcogens has been rarely explored. In a recent study, we demonstrated site-selective oxygen-to-sulfur substitution in the Keggin-type POM [SiW_12_O_40_]^4−^. Building on this, we now report the first synthesis of a polyoxoselenidotungstate, featuring terminal selenido ligands (W=Se bonds), using a site-selective oxygen-to-selenium substitution reaction. By reacting [SiW_12_O_40_]^4−^ with Woollins' reagent (2,4-diphenyl-1,3,2,4-diselenadiphosphetane 2,4-diselenide) in organic solvents, all twelve terminal oxido ligands (W=O) were selectively converted to selenido ligands (W=Se). The resulting compound [SiW_12_O_28_Se_12_]^4−^ retains the Keggin-type framework and exhibits distinct optical and electronic properties owing to the incorporated selenium atoms. These findings pave the way for the systematic modification of oxygen sites in POMs with the heavier chalcogens sulfur and selenium, opening new avenues for tailoring their properties and expanding their utility across diverse fields of materials science.

## Abstract 2

We report the first synthesis of [SiW_12_O_28_Se_12_]^4−^*via* site-selective oxygen-to-selenium substitution in [SiW_12_O_40_]^4−^. The properties are controllable by varying 12 terminal atoms (O, S, or Se).

## Graphical abstract

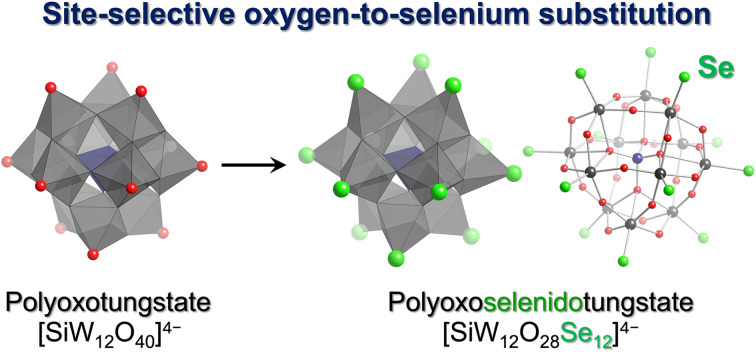

## Introduction

Metal chalcogenides (*i.e.* oxides, sulfides, and selenides) represent a versatile class of semiconductive materials with diverse properties and resulting applications spanning catalysis, optics, electrochemistry, energy storage, sensing, and medicine.^1,2^ Their structures and constituent chalcogen elements play a critical role in determining their properties and applications.^2^ In particular, the unoccupied 3d orbital of a selenium atom can contribute to bonding with metal atoms, which often imparts electron storage/transportation and catalytic properties.^3^ However, among the wide range of metal chalcogenide clusters,^3*c*–*f*^ selenides are less commonly explored compared to oxides and sulfides, underscoring the need for efficient and precise synthetic approaches for selenide-containing cluster compounds.

Polyoxometalates (POMs) are anionic molecular metal oxides of the early transition metals (W^VI^, Mo^VI^, V^V^, Nb^V^, and Ta^V^). They display well-defined structures with tunable properties such as acidity/basicity, redox behavior, and photochemical activity, enabling their broad applications in catalysis, medicine, energy storage and conversion, sensing, electronics, and battery technologies.^4^ Their properties can be tailored by altering their structures, atomic composition, or electronic states. To date, various metal atoms, metal oxide clusters, and metal nanoclusters have been incorporated into POM frameworks by substitution of their metal sites or by derivatization of lacunary POMs containing vacant metal sites.^5–7^ While organic ligands have been widely substituted for oxygen sites in POMs,^8,9^ anion-substituted POMs, *i.e.* where oxygen atoms are replaced by simple anions, such as halides or chalcogenides, have garnered relatively less attention.^10^ To date, several sulfido (S^2−^)-containing POMs have been synthesized *via* aggregation of small cationic metal sulfide species (*e.g.* [M_2_O_2_S_2_]^2+^; M = Mo^V^, W^V^), where sulfur atoms serve as bridging sites (*i.e*. M–S–M; M = Mo^V^, W^V^),^11^ or by replacing terminal oxido ligands (O^2−^) on Nb or Ta atoms in [(O=M)PW_11_O_39_]^4−^ and [(O=M)W_5_O_18_]^3−^(M = Nb^V^, Ta^V^) with sulfido ligands (S^2−^).^12^ In contrast, reports on POMs possessing selenido ligands (Se^2−^) remain scarce, likely due to their low stability. Only two reports have been documented: Keplerate-type (M_72_Mo^V^_60_Se_60_; M = Mo^VI^, W^VI^) featuring bridging selenido ligands (*i.e*. Mo^V^–Se^2−^–Mo^V^),^13^ Keggin-type [(Se=M)PW_11_O_39_]^4−^ (M = Nb^V^, Ta^V^) and the Lindqvist anion [(Se=Nb)W_5_O_18_]^3−^ where terminal selenido ligands are coordinated to a niobium or tantalum site.^14^

Recently, we reported site-selective oxygen-to-sulfur substitution reactions in a series of Keggin-type POMs, [XW_12_O_40_]^*n*−^ (X = Al^III^, Si^IV^, Ge^IV^, and P^V^), successfully yielding polyoxosulfidotungstates (also known as polyoxothiometalates), [XW_12_O_28_S_12_]^*n*−^.^15,16^ In these transformations, sulfurizing agents selectively replaced all twelve terminal oxido ligands of [XW_12_O_40_]^*n*−^ with sulfido ligands while retaining the characteristic Keggin-type structure. The resulting [XW_12_O_28_S_12_]^*n*−^ compounds exhibited distinctive visible-light absorption characteristics, electronic structures, and electrochemical properties. Notably, the [XW_12_O_28_S_12_]^*n*−^ compounds could not be synthesized through the conventional dehydrative condensation of oxo(thio)anions (*e.g.* [WS_*x*_O_4−*x*_]^2−^), a common route in POM synthesis. This indicates that [XW_12_O_28_S_12_]^*n*−^ can only be obtained *via* site-selective oxygen-to-sulfur substitution. These findings underscore the potential of site-selective anion substitution as a powerful strategy for introducing unique structures, properties, and functions into POMs—although its application has thus far been limited to sulfur substitution. Given that selenium, like oxygen and sulfur, is a group 16 element, it should be capable of incorporation into POM frameworks *via* site-selective substitution, potentially enabling the discovery of previously unreported selenium-containing POMs, specifically polyoxoselenidotungstates.

**Fig. 1:**
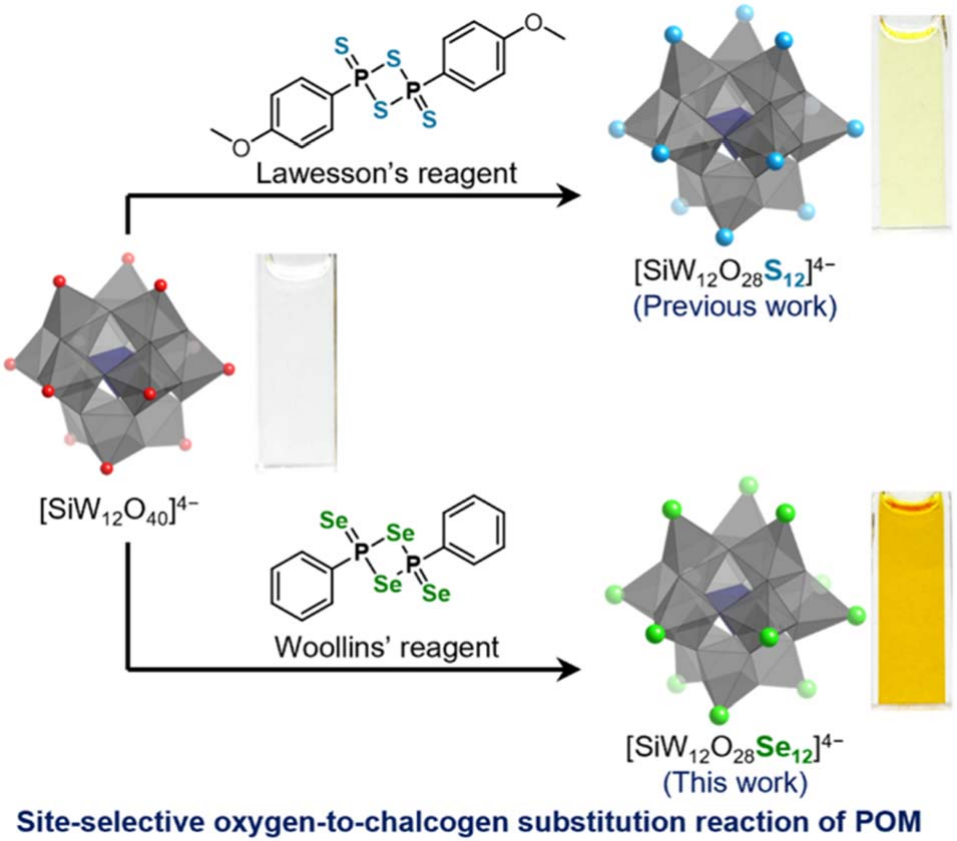
Schematic representation of syntheses of Keggin-type polyoxosulfidotungstate ([SiW_12_O_28_S_12_]^4−^; previous work)^15^ and polyoxoselenidotungstate ([SiW_12_O_28_Se_12_]^4−^; this work) through the site-selective oxygen-to-chalcogen substitution of Keggin-type polyoxotungstate [SiW_12_O_40_]^4−^, and the photographs of 1 mM acetonitrile solutions of tetra-*n*-butylammonium (TBA) salts of each polyanion (*i.e*. (TBA)_4_[SiW_12_O_28_E_12_] (E = O, S, and Se)).

Herein, we report the synthesis of the Keggin-type polyoxoselenidotungstate [SiW_12_O_28_Se_12_]^4−^ through a site-selective oxygen-to-selenium substitution of [SiW_12_O_40_]^4−^ (Fig. 1). All twelve terminal oxygen atoms in [SiW_12_O_40_]^4−^ were replaced by selenium atoms. This study demonstrates the first successful synthesis of a polyoxoselenidotungstate featuring terminal W=Se bonds. Selenium incorporation results in notable changes in the optical properties and electronic structure of [SiW_12_O_28_Se_12_]^4−^ compared to those of both [SiW_12_O_40_]^4−^ and known polyoxosulfidotungstates [XW_12_O_28_S_12_]^*n*−^. These findings indicate that site-selective oxygen-to-chalcogen substitution offers a precise route for the post-synthetic modification of POM structures and properties. This approach promotes the high-throughput design of POM-based, selenium-containing materials, enhancing their potential and broadening their application range.

## Results and discussion

### Synthesis of Keggin-type [SiW_12_O_28_Se_12_]^4−^

We first explored the site-selective oxygen-to-selenium substitution of [SiW_12_O_40_]^4−^ by reacting its tetra-*n*-butylammonium (TBA) salt with selenium-containing reagents. Previously, we reported that 1,3,2,4-dithiadiphosphetane-2,4-dithione derivatives (R–P_2_S_4_–R),^17^ such as Lawesson's reagent (*i.e.*, R = –C_6_H_4_OMe)^15^ act as effective sulfurizing agents for the site-selective oxygen-to-sulfur substitution of [SiW_12_O_40_]^4−^ (Fig. 1). In contrast, other sulfur-based reagents, including bis(trimethylsilyl)sulfide, tetraphosphorous decasulfide (P_4_S_10_), triphenylphosphine sulfide, and dimethyl trisulfide, exhibit little to no reactivity toward [SiW_12_O_40_]^4−^. Building upon these findings, we used Woollins' reagent (2,4-diphenyl-1,3,2,4-diselenadiphosphetane 2,4-diselenide)^18^ that possesses an analogous four-membered ring structure with terminal selenido ligands (Fig. 1). Taking into account the solubilities of both (TBA)_4_[SiW_12_O_40_] and Woollins' reagent we selected a mixed solvent of acetonitrile/1,2-dichloroethane for the reaction. At room temperature (∼25 °C), the reaction of (TBA)_4_[SiW_12_O_40_] and Woollins' reagent (seven equivalents with respect to (TBA)_4_[SiW_12_O_40_]) proceeded minimally, as indicated by electrospray ionization mass (ESI-mass) spectrometry and the negligible color change of the reaction solution. However, heating the mixture to 60 °C for 20 h caused the colorless solution of (TBA)_4_[SiW_12_O_40_] to turn dark red (see the ESI† for details). The final red crystalline product was obtained by washing with dichloromethane and recrystallizing from a mixed solvent of acetonitrile and diethyl ether (47% yield).

**Fig. 2:**
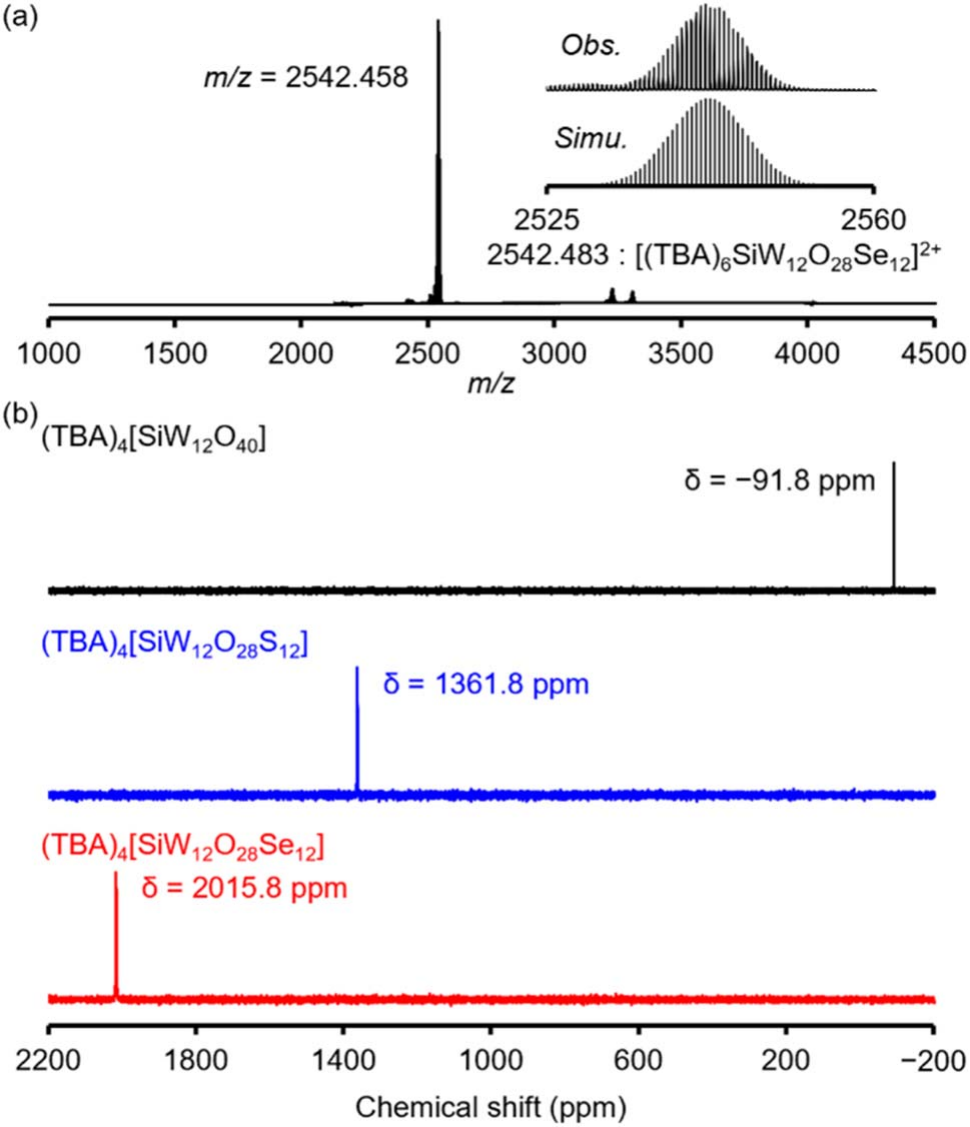
(a) ESI-mass spectrum of (TBA)_4_[SiW_12_O_28_Se_12_] in acetonitrile (positive ionization mode). (b) ^183^W NMR spectra of (TBA)_4_[SiW_12_O_40_] (black), (TBA)_4_[SiW_12_O_28_S_12_] (blue), and (TBA)_4_[SiW_12_O_28_Se_12_] (red) in dimethylsulfoxide-*d*_6_.

The ESI-mass spectrum of the product dissolved in acetonitrile displayed a signal at *m*/*z* = 2542.458 (*z* = 2), corresponding to [(TBA)_6_SiW_12_O_28_Se_12_]^2+^ (theoretical *m*/*z* = 2542.483) (Fig. 2a). This result indicates the formation of the [SiW_12_O_28_Se_12_]^4−^ anion, wherein all 12 oxygen atoms of [SiW_12_O_40_]^4−^ are replaced by selenium atoms. Elemental analysis further confirmed the molecular formula of the product to be (TBA)_4_[SiW_12_O_28_Se_12_]. In addition, the ESI-mass spectrum of (TBA)_4_[SiW_12_O_28_Se_12_] showed no significant change over one week in an acetonitrile solution containing 1 vol% water (*ca.* 10 000 equivalents with respect to (TBA)_4_[SiW_12_O_28_Se_12_]), indicating that (TBA)_4_[SiW_12_O_28_Se_12_] exhibits high stability under these conditions (Fig. S1†).

The ^183^W nuclear magnetic resonance (NMR) spectrum of (TBA)_4_[SiW_12_O_28_Se_12_] in dimethylsulfoxide-*d*_6_ showed a single signal, indicating that all twelve W atoms are equivalent and (TBA)_4_[SiW_12_O_28_Se_12_] was obtained in high purity (Fig. 2b). Notably, the ^183^W NMR signal of (TBA)_4_[SiW_12_O_28_Se_12_] (*δ* = 2015.8 ppm) appeared at a significantly lower field compared to those of (TBA)_4_[SiW_12_O_40_] (*δ* = −91.8 ppm) and (TBA)_4_[SiW_12_O_28_S_12_] (*δ* = 1361.8 ppm). Density functional theory (DFT) was used to calculate ^183^W NMR chemical shifts. The computed shifts for (TBA)_4_[SiW_12_O_28_Se_12_] (*δ*_cal_ = 1805 ppm) and (TBA)_4_[SiW_12_O_28_S_12_] (*δ*_cal_ = 1239 ppm) were in excellent agreement with experimental data, confirming the significant differences in the ^183^W NMR chemical shift between these compounds. Furthermore, the calculated shift for (TBA)_4_[SiW_12_O_40_] (*δ*_cal_ = −62 ppm) was consistent with its expected upfield position relative to (TBA)_4_[SiW_12_O_28_Se_12_] and (TBA)_4_[SiW_12_O_28_S_12_]. Despite the wide chemical shift range observed (≈2100 ppm), the ^183^W NMR calculations strongly support the experimental results.

It is known that ^183^W NMR chemical shifts are largely governed by the inverse of the energy gap between the unoccupied and occupied molecular orbitals. Considering the frontier orbital diagram (Fig. S2 and S3†), this involves the energy difference between occupied W^VI^–ligand bonding orbitals with a predominant ligand component and unoccupied W^VI^–ligand antibonding orbitals with a predominant metal component. As the ligand varies among O^2−^, S^2−^, and Se^2−^, the energies of the occupied orbitals increase relative to the predominantly metal based unoccupied orbitals. This leads to a reduction of the energy gap as the electronegativity of the ligand decreases (see the optical and electrochemical properties section), resulting in a downfield shift in the ^183^W NMR spectrum.^19^ Therefore, the observed downfield shift of (TBA)_4_[SiW_12_O_28_Se_12_] relative to (TBA)_4_[SiW_12_O_28_S_12_] and (TBA)_4_[SiW_12_O_40_] is consistent with the trend in chalcogen electronegativity: O (3.44) > S (2.58) > Se (2.55).

### Structures of [SiW_12_O_28_E_12_]^4−^ (E = O, S, and Se)

**Fig. 3:**
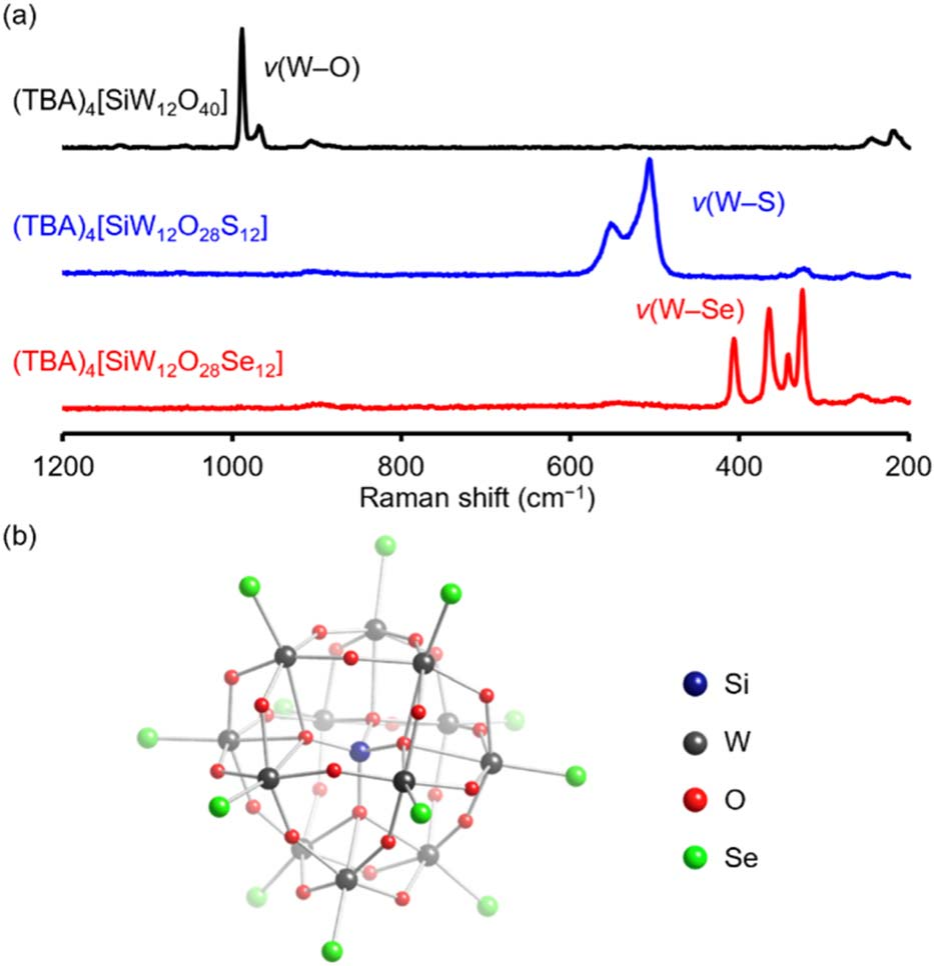
(a) Raman spectra of (TBA)_4_[SiW_12_O_40_] (black), (TBA)_4_[SiW_12_O_28_S_12_] (blue), and (TBA)_4_[SiW_12_O_28_Se_12_] (red). (b) Crystal structure of the anionic component of the tetraphenylphosphonium (TPP) salts of [SiW_12_O_28_Se_12_]^4−^. Color code: dark blue, Si; black, W; red, O; light green, Se.

In the Raman spectrum of (TBA)_4_[SiW_12_O_40_], two peaks appearing at 968 and 989 cm^−1^ were assigned to the stretching vibrations of terminal W=O bonds.^15,20^ In contrast, the Raman spectrum of (TBA)_4_[SiW_12_O_28_Se_12_] displayed no prominent peaks associated with W=O bonds but instead presented four intense peaks at 326, 342, 365, and 407 cm^−1^, closely resembling the pattern observed for the [WSe_4_]^2−^ anion (Fig. 3a and Table S1†).^21^ The observed wavenumbers were significantly lower than those reported for terminal W=S stretching vibrations (506 and 550 cm^−1^) in (TBA)_4_[SiW_12_O_28_S_12_],^15^ supporting the incorporation of heavier selenium atoms in place of oxygen or sulfur atoms. Additionally, the infrared spectrum of (TBA)_4_[SiW_12_O_28_Se_12_] displayed two peaks at 349 and 327 cm^−1^, likely attributable to stretching vibrations of W=Se bonds (Fig. S4†). These results confirm that all twelve terminal oxido ligands in (TBA)_4_[SiW_12_O_40_] were selectively replaced by selenido ligands through the reaction with Woollins' reagent.

To further investigate the structure, we conducted X-ray crystallographic analysis of [SiW_12_O_28_Se_12_]^4−^ after replacing the TBA counterions with tetraphenylphosphonium (TPP). The crystallographic data confirmed that four TPP cations replaced the original four TBA cations. In the parent Keggin-type polyoxotungstate structure, the 40 oxygen atoms fall into three categories: four μ_4_-oxygen atoms surrounding the central silicon atom, 24 μ_2_-oxygen atoms bridging tungsten atoms (W–O–W), and 12 terminal oxygen atoms (W=O).^22^ The anionic structure of [SiW_12_O_28_Se_12_]^4−^ (Fig. 3b and Table S2†) retains the α-Keggin-type framework, with all terminal W=O oxido ligands in [SiW_12_O_40_]^4−^ (Table S2 and S3†) replaced by terminal W=Se selenido ligands, while the other oxygen atoms, comprising μ_4_-O and μ_2_-O ligands, remain unaltered. These findings confirm that Woollins' reagent enables site-selective substitution of the terminal oxygen atoms in [SiW_12_O_40_]^4−^ with selenium, consistent with the results of ESI-mass and Raman spectral results. Bond valence sum (BVS) values for silicon (3.99, 4.04) and tungsten (6.22–6.42) in [SiW_12_O_28_Se_12_]^4−^ indicate that their oxidation states remain at +4 and +6, respectively (Table S4†), closely matching those in [SiW_12_O_40_]^4−^ (Table S3†). BVS values of selenium atoms range from 1.62 to 2.00 (Table S5†) and confirm their identity as selenido (W=Se) rather than hydrogen selenido (W–SeH) ligands, consistent with the molecular formula (TPP)_4_[SiW_12_O_28_Se_12_].

A comparison of the crystal structures of [SiW_12_O_40_]^4−^, [SiW_12_O_28_S_12_]^4−^, and [SiW_12_O_28_Se_12_]^4−^ reveals a systematic increase in the average W=E bond length (E = O, S, and Se): 1.71 Å for W=O in [SiW_12_O_40_]^4−^, 2.15 Å for W=S in [SiW_12_O_28_S_12_]^4−^,^15^ and 2.28 Å for W=Se in [SiW_12_O_28_Se_12_]^4−^ (Table S6†). This trend correlates well with the increasing ionic radii of O^2−^, S^2−^, and Se^2−^.^23^ Notably, the bond lengths of Si–μ_4_-O, W–μ_4_-O, and W–μ_2_-O remain nearly unchanged across [SiW_12_O_40_]^4−^, [SiW_12_O_28_S_12_]^4−^, and [SiW_12_O_28_Se_12_]^4−^, indicating that oxygen-to-chalcogen substitution occurs selectively at the terminal sites without substantially affecting the internal framework.

To date, only two reports have described the synthesis of POMs featuring M=Se bonds *i.e*. [(Se=M)PW_11_O_39_]^4−^ (M = Nb^V^, Ta^V^) and [(Se=Nb)W_5_O_18_]^3−^.^14^ The Keggin-type [SiW_12_O_28_Se_12_]^4−^ anion is the first example of a polyoxoselenidotungstate containing terminal selenium atoms bonded to W^VI^. The W^VI^=Se bond remains largely unexplored, with prior observations limited to tetrahedral [WSe_4_]^2−^ and its derivatives^24^ and a square-pyramidal {WSe_5_} structure.^25^ To our knowledge, polyoxoselenidotungstate [SiW_12_O_28_Se_12_]^4−^ is the first molecular structure wherein terminal selenido ligands are coordinated to octahedral W^VI^.

### Optical and electrochemical properties

**Fig. 4:**
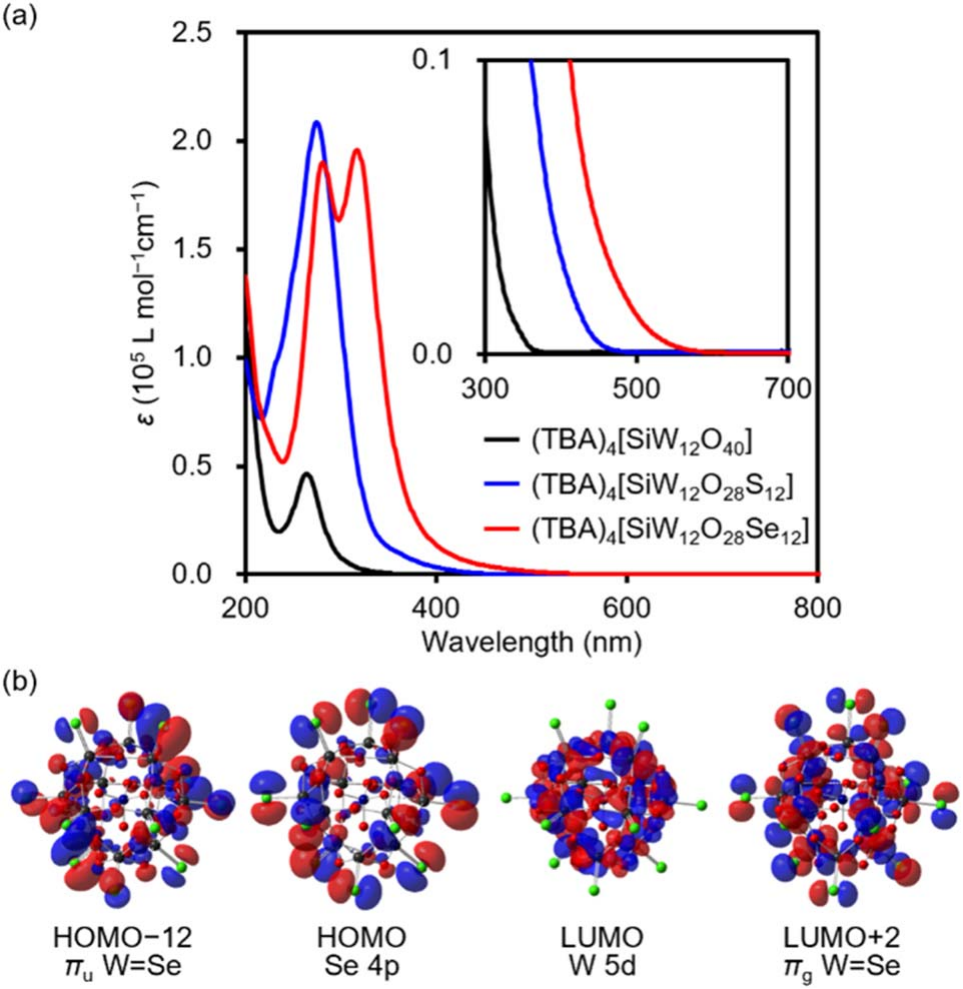
(a) UV-vis spectra of (TBA)_4_[SiW_12_O_40_] (10 μM, black), (TBA)_4_[SiW_12_O_28_S_12_] (5 μmol L^−1^, blue),^15^ and (TBA)_4_[SiW_12_O_28_Se_12_] (8 μmol L^−1^, red) in acetonitrile. Inset: enlarged view. (b) Selected molecular orbitals of the [SiW_12_O_28_Se_12_]^4−^ anion based on DFT calculations.

The UV-vis spectrum of (TBA)_4_[SiW_12_O_28_Se_12_] in acetonitrile displays two intense absorption bands at *λ* = 281 nm (*ε* = 1.9 × 10^5^ L mol^−1^ cm^−1^) and *λ* = 317 nm (*ε* = 2.0 × 10^5^ L mol^−1^ cm^−1^) (Fig. 4a). These bands are red-shifted compared to those of (TBA)_4_[SiW_12_O_40_] (*λ* = 264 nm; *ε* = 4.6 × 10^4^ L mol^−1^ cm^−1^) and (TBA)_4_[SiW_12_O_28_S_12_] (*λ* = 271 nm; *ε* = 2.1 × 10^5^ L mol^−1^ cm^−1^).^15^ Additionally, the absorption tail of (TBA)_4_[SiW_12_O_28_Se_12_] extends to approximately 580 nm, significantly extended to longer wavelengths than those of (TBA)_4_[SiW_12_O_40_] (*λ* = *ca.* 370 nm) and (TBA)_4_[SiW_12_O_28_S_12_]^4−^ (*λ* = *ca.* 470 nm), indicating a substantial alteration in the electronic structure. Consequently, the acetonitrile solution of (TBA)_4_[SiW_12_O_28_Se_12_] appears orange, whereas those of (TBA)_4_[SiW_12_O_28_S_12_] and (TBA)_4_[SiW_12_O_40_] are pale yellow and colorless, respectively (Fig. 1).

To further investigate the electronic structure of [SiW_12_O_28_Se_12_]^4−^, we performed DFT calculations. In compound [SiW_12_O_40_]^4−^, the lowest unoccupied molecular orbital (LUMO) is primarily composed of W 5d orbitals, while the highest occupied molecular orbital (HOMO) is mainly derived from μ_2_-O 2p orbitals.^15^ In contrast, the LUMO and LUMO+1 levels of [SiW_12_O_28_Se_12_]^4−^ are primarily composed of W 5d orbitals, while the occupied orbitals include contributions from Se 4p orbitals (HOMO to HOMO−11), W–Se bonding π-orbitals (HOMO−12 to HOMO−23), and W–Se bonding σ-orbitals (HOMO−24 to HOMO−26). These orbitals are higher in energy than the μ_2_-O 2p orbitals, such as HOMO−27 (Fig. 4b and S2†). Owing to the presence of these Se-derived occupied orbitals, the HOMO–LUMO gap of [SiW_12_O_28_Se_12_]^4−^ (5.73 eV) is substantially smaller than that of [SiW_12_O_40_]^4−^ (6.85 eV) and slightly smaller than that of [SiW_12_O_28_S_12_]^4−^ (5.86 eV).^15^ DFT calculations also reveal the formation of unoccupied W–Se antibonding π orbitals (*e.g.*, LUMO+2 to LUMO+8). Based on the time-dependent DFT calculations, the absorption bands at *λ* = 281 and 317 nm in (TBA)_4_[SiW_12_O_28_Se_12_] were likely attributed to charge-transfer transitions from W–Se bonding π-orbitals (*i.e*. HOMO−12 to HOMO−23) and Se 4p orbitals (*i.e*. HOMO to HOMO−11) to W–Se antibonding π orbitals and W 5d orbitals (*i.e*. LUMO+9 to LUMO+11) (Fig. S5†). These results demonstrate the strong influence of selenium substitution on the electronic states and optical properties of POMs.

**Fig. 5:**
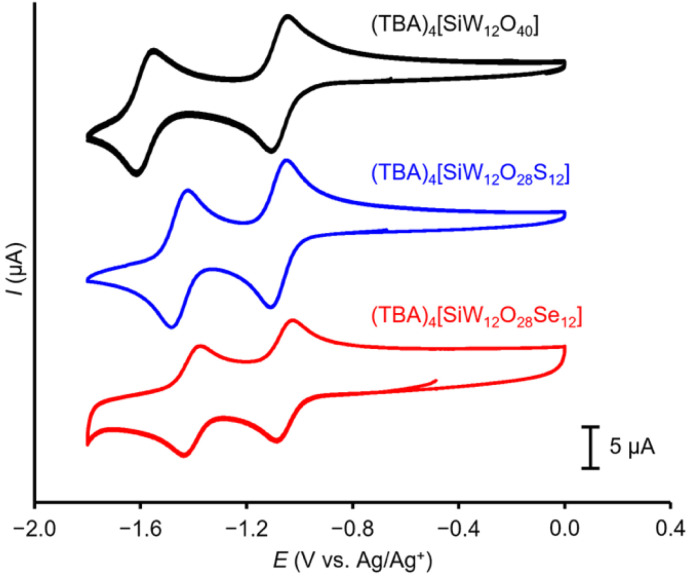
Cyclic voltammograms of (TBA)_4_[SiW_12_O_40_] (black line), (TBA)_4_[SiW_12_O_28_S_12_] (blue line), and (TBA)_4_[SiW_12_O_28_Se_12_] (red line) in acetonitrile containing (TBA)ClO_4_ (0.10 mol L^−1^; 100 mV s^−1^).

Finally, to investigate the electrochemical properties of a series of (TBA)_4_[SiW_12_O_28_E_12_] (E = O, S, and Se), we carried out the cyclic voltammetry measurements in acetonitrile containing (TBA)ClO_4_ (0.10 mol L^−1^; Fig. 5). The cyclic voltammograms of (TBA)_4_[SiW_12_O_28_E_12_] showed reversible redox behavior. The first redox wave of (TBA)_4_[SiW_12_O_28_Se_12_] appeared at −1.08 V *vs.* Ag/Ag^+^, similar to those observed for (TBA)_4_[SiW_12_O_40_] and (TBA)_4_[SiW_12_O_28_S_12_].^15,16^ In contrast, the second reduction wave showed a distinct trend, with the reduction potential shifting positively in the order of E = O (−1.62 V), S (−1.48 V), and Se (−1.43 V).

## Conclusions

This study demonstrates the synthesis of a polyoxoselenidotungstate through the site-selective substitution of terminal oxido ligands (W=O) in the parent polyoxotungstate with selenido ligands (W=Se) using Woollins' reagent. The resulting selenium-containing polyoxoselenidotungstate, [SiW_12_O_28_Se_12_]^4−^, retains the α-Keggin-type framework of its precursor, [SiW_12_O_40_]^4−^ and exhibits high stability in acetonitrile, while exhibiting a distinct electronic structure. Specifically, [SiW_12_O_28_Se_12_]^4−^ features molecular orbitals derived from Se 4p and W–Se bonding/antibonding orbitals, leading to extended absorption into the visible-light region (up to *ca.* 580 nm), in contrast to [SiW_12_O_40_]^4−^ and the previously reported [SiW_12_O_28_S_12_]^4−^. This study expands the range of chalcogen-substituted metal oxides, demonstrating precise oxygen-to-selenium substitution at the terminal sites. Such structural modifications open new avenues for tailoring the electronic properties of POM-based materials, with potential applications in (photo)catalysis, sensing, optics, energy conversion, and battery technologies.

## Author contributions

K. Yonesato and K. S. designed the project and experiments. K. Yonesato performed the major part of experiments. Y. W. contributed to the ^183^W NMR measurements and manuscript revision. M. P.-B. and R. J. E. contributed to the ^183^W NMR measurements, calculations and manuscript revision. K. Yonesato, K. S., and K. Yamaguchi cowrote the manuscript.

## Conflicts of interest

There are no conflicts to declare.

## Supplementary Material

SC-016-D5SC03340C-s001

SC-016-D5SC03340C-s002

## Data Availability

The data supporting this manuscript are available in the ESI and available on request. Crystallographic data for TPP salts of [SiW_12_O_40_]^4−^ and [SiW_12_O_28_Se_12_]^4−^ have been deposited at the CCDC (deposition numbers 2440303 and 2440307).
