# Supplementary material for: Synthesis of a Keggin-type polyoxoselenidotungstate via site-selective oxygen-to-selenium substitution
Source: Chem Sci. 2025 Jun 16;16(29):13183–8. doi: 10.1039/d5sc03340c (PMC12183570; doi:10.1039/d5sc03340c)
Supplement: SC-016-D5SC03340C-s001 [file SC-016-D5SC03340C-s001.pdf]

Supplementary Information (SI) for Chemical Science.
This journal is © The Royal Society of Chemistry 2025

Electronic Supplementary Information (ESI) for

Synthesis of a Keggin-type polyoxoselenidotungstate via site-selective oxygen-to-selenium substitution

Kentaro Yonesato,*[a] Yota Watanabe,[a] Magda Pascual-Borràs,[b] R. John Errington,[b] Kazuya Yamaguchi[a] and Kosuke Suzuki *[a,c]

[a] Department of Applied Chemistry, School of Engineering, The University of Tokyo, 7-3-1 Hongo, Bunkyo-ku, Tokyo 113-8656 Japan

[b] NUPOM Lab, Chemistry, School of Natural & Environmental Sciences, Newcastle University, NE1 7RU Newcastle Upon Tyne, UK

[c] Department of Advanced Materials Science, Graduate School of Frontier Sciences, The University of Tokyo, 5-1-5 Kashiwanoha, Kashiwa, Chiba, 277-8561 Japan

E-mail: ksuzuki@appchem.t.u-tokyo.ac.jp; k-yonesato@g.ecc.u-tokyo.ac.jp

Contents

Experimental section S2–S4

Table S1–S4 S5–S6

Figures S1–S5 S7–S10

References S11

Experimental Section

Material:

Acetonitrile, 1,2-dichloroethane, dichloromethane, and diethyl ether were purchased from Kanto Chemical Co., Inc. and stored with appropriate molecular sieves. Woollins' reagent (2,4-diphenyl-1,3,2,4-diselenadiphosphetane 2,4-diselenide) was obtained from Merck Sigma-Aldrich. $H_4SiW_{12}O_{40}$ was purchased from FUJIFILM Wako Chemicals. Tetra-*n*-butylammonium bromide (TBA)Br and tetraphenylphosphonium bromide (TPP)Br were obtained from Tokyo Chemical Industry. $(TBA)_4[SiW_{12}O_{40}]$ and $(TBA)_4[SiW_{12}O_{28}S_{12}]$ were synthesized according to the reported procedure.[S1,S2]

Instruments

Elemental analyses for carbon, hydrogen, and nitrogen were performed on a MICRO CORDER JM10, HSU 20, and ICS-1100 at the Core Facility Center, Science Tokyo. Ultraviolet–visible absorption spectra were measured using a JASCO V-770 with a 1 cm quartz cell at room temperature (~25 °C). IR spectra were measured in transmission mode on a JASCO FT/IR-4100 spectrometer using KBr disks. Raman spectra were measured on a JASCO NRS-5100 spectrometer under an irradiation laser of wavelength λ = 532 nm (0.1 mW). ESI-mass spectra were recorded on a Shimadzu LCMS-9050 instrument and a Waters Xevo G2-XS QTof instrument. ^{183}W NMR (20.84 MHz) spectra were recorded using a JEOL ECA-500 spectrometer by using 10 mm tubes. Chemical shifts (δ) were reported in parts per million (ppm) downfield from 1 M Na_2WO_4 (solvent, D_2O). Cyclic voltammetry measurements were performed with a BioLogic VSP-300 at the scan rate of 100 mV s^{-1}. A standard three-electrode arrangement was employed with a glassy carbon disk electrode as the working electrode and a platinum wire as the counter electrode. The potentials were measured using $Ag/AgNO_3$ reference electrode (10 mM $AgNO_3$, 100 mM $TBAClO_4$ in acetonitrile).

Synthesis of $(TBA)_4[SiW_{12}O_{28}Se_{12}]$

This experiment was carried out under air in the fume hood due to the bad smell of Woollins' reagents and byproducts. To a 30 mL mixed solvent of acetonitrile/1,2-dichloroethane (1/1, v/v), $(TBA)_4[SiW_{12}O_{40}]$ (1.0 g, 0.26 mmol) and Woollins' reagent (1.00 g, 1.87 mmol) were added, and the solution was stirred for a day at 60°C. After filtration, the resultant red solution was added dropwise to an excess of diethyl ether (600 mL). The orange precipitate formed was collected by filtration. The orange powder of $(TBA)_4[SiW_{12}O_{28}Se_{12}]$ was obtained after washing with excess dichloromethane (783 mg, 65% yield). The resultant crude sample of $(TBA)_4[SiW_{12}O_{28}Se_{12}]$ was further purified by recrystallization from a mixture of acetonitrile and diethyl ether (561 mg; 47% yields, orange block crystals).

Positive-ion MS (ESI, acetonitrile): *m/z* 2542.592 ($[(TBA)_6SiW_{12}O_{28}Se_{12}]^{2+}$; theoretical *m/z* = 2542.483). IR (KBr pellet, cm^{-1}) 3438, 2959, 2928, 2871, 1631, 1478, 1378, 1105, 1058, 1008, 966, 933, 882, 858, 766, 531, 379, 349, 327. Elemental analysis calcd (%) for $(TBA)_4[SiW_{12}O_{28}Se_{12}]$: C, 16.71; H, 3.16; N, 1.22. Found: C, 16.52; H, 3.14; N, 1.30. UV-vis (acetonitrile solution): λ (ε) 281 nm (1.9×10^5 L mol^{-1} cm^{-1}), 317 nm (2.0×10^5 L mol^{-1} cm^{-1}).

X-ray crystallography

The crystallographic analyses of $[SiW_{12}O_{40}]^{4-}$ and $[SiW_{12}O_{28}Se_{12}]^{4-}$ were carried out on their TPP salts. The crystals of TPP salts of $[SiW_{12}O_{40}]^{4-}$ and $[SiW_{12}O_{28}Se_{12}]^{4-}$ suitable for crystallography were obtained by cation exchange reactions. The solutions of $(TBA)_4[SiW_{12}O_{40}]$ or $(TBA)_4[SiW_{12}O_{28}Se_{12}]$ (~1 mg) in acetonitrile (1 mL) were allowed to stand overnight after addition of acetonitrile solutions containing (TPP)Br (6 equivalents with respect to $TBA_4[SiW_{12}O_{40}]$ or 4 equivalents with respect to $TBA_4[SiW_{12}O_{28}Se_{12}]$). The single-crystal X-ray diffraction experiment of $(TPP)_4[SiW_{12}O_{40}]$ was made on Rigaku XtaLab Synergy-R diffractometer equipped with a HyPix-6000HE detector and rotating-anode with Mo Kα radiation (λ = 0.71073 Å, 50 kV, 24 mA) at 178 K. The diffraction experiment of $(TPP)_4[SiW_{12}O_{28}Se_{12}]$) was carried out using the BL02B1 beamline at SPring-8 facility of the Japan Synchrotron Radiation Research Institute with a PILATUS3 X CdTe 1M detector at 100 K. The incident X-ray beam (λ = 0.4132 Å) was monochromatized by a Si(311) double-crystal monochromator. The data collection and process were conducted using RAPID AUTO and CrysAlisPro software,[S3] respectively. In the reduction of data, Lorentz, polarization, and empirical absorption corrections were made. The structural analyses were performed using Olex[2] and WinGX.[S4,S5] Structures were solved using SHELXT-2018/2 (intrinsic phasing methods) and refined by SHELXL-2018/3.[S6,S7] All non-hydrogen atoms (C, O, Si, P, S, and W) were refined anisotropically. CCDC-2440303 and 2440307 contain the supplementary crystallographic data of $(TPP)_4[SiW_{12}O_{40}]$ and $(TPP)_4[SiW_{12}O_{28}Se_{12}]$, respectively. The data can be obtained free of charge from The Cambridge Crystallographic Data Centre via www.ccdc.cam.ac.uk/data_request/cif.

Bond valence sum (BVS) calculations

The BVS values were calculated by the expression for the variation of the length r_{ij} of a bond between two atoms i and j in observed crystal with valence Vi.

$$V_i = \sum \exp\left(\frac{r'_0 - r_{ij}}{B}\right)$$

where B is constant equal to 0.37 Å, r'_0 is bond valence parameter for a given atom pair.[S8]

DFT calculations

DFT calculations were performed using Gaussian 16, Rev. B.01. The geometries used in the calculation were based on the crystal structures determined in this study. The anion structure of $[SiW_{12}O_{28}Se_{12}]^{4-}$ was optimized at the CAM-B3LYP functional with 6-31G(d) for Si, O and Se, and LanL2DZ for W by using the polarizable continuum model with the parameters of the integral equation formalism model for acetonitrile.

DFT calculations for ^{183}W NMR chemical shifts were performed using ADF2022 package.[S9-S11] The process for obtaining the ^{183}W NMR chemical shifts consists of (i) a geometry optimization step and (ii) a single-point NMR calculation. The geometries were optimised with all-electron triple-ζ+ double polarization (TZ2P) Slater- type basis sets with the GGA-type PBE[S12] functional. For NMR calculations, we used all-electron triple-ζ+ polarization (TZP) Slater- type basis set and OPBE[S13] functional with spin–orbit (SO) relativistic corrections to the electrons via the zeroth-order regular approximation (ZORA).[S14-S16] This methodology was already reported to be the best to reproduce ^{183}W NMR in polyoxometalates.[S17] Since were dealing with anionic species in solution, we applied the effects of solvent and counterions as a continuum via the conductor-like screening model (COSMO), with a given dielectric constant (ε) that induces charge polarization on a surface around the molecule.[S18-S19] Taking the optimized geometry, the NMR single-point calculation is done for the target and reference (WO_4^{2-}) compounds introducing spin-orbit (SO) corrections and the GIAO method.[S20-S22] The calculated chemical shift is determined as $\delta_{cal} = \sigma_{ref} - \sigma_x$, where σ_x and σ_{ref} are the isotropic average shielding for the nucleus of the target and the reference compounds, respectively.

Table S1. Raman shift (cm^{-1}) of ν(W–E) vibrations of $(TBA)_4[SiW_{12}O_{28}E_{12}]$ (E = O, S, and Se).

Compound	Raman shift (cm^{-1}) of ν(W–E) vibration			
$(TBA)_4[SiW_{12}O_{40}]$	968	989		
$(TBA)_4[SiW_{12}O_{28}S_{12}]$	506	550		
$(TBA)_4[SiW_{12}O_{28}Se_{12}]$	326	342	365	407

Table S2. Crystallographic parameters of $(TPP)_4[SiW_{12}O_{40}]$ and $(TPP)_4[SiW_{12}O_{28}Se_{12}]$.

	$(TPP)_4[SiW_{12}O_{40}]$	$(TPP)_4[SiW_{12}O_{28}Se_{12}]$
Crystal system	Triclinic	Triclinic
Space group	*P*1 (#1)	*P*1 (#1)
Formula	$C_{104}H_{92}N_4P_4SiW_{12}O_{40}$	$C_{100}H_{86}N_2P_4SiW_{12}O_{28}Se_{12}$
Fw (g mol^{-1})	4395.98	5069.39
a (Å)	13.90582(11)	13.76778(10)
b (Å)	15.07813(13)	14.64503(10)
c (Å)	15.90307(14)	17.18784(13)
α (deg)	112.3714(8)	68.0094(7)
β (deg)	104.2936(7)	68.5531(7)
γ (deg)	100.2025(7)	89.6202(6)
V (Å^3)	2848.02(5)	2956.65(4)
Z	1	1
ρ_{calc} (g cm^{-3})	2.563	2.847
Temp (K)	178(2)	100(2)
Flack parameter	0.251(4)	0.385(12)
R_1 [$I > 2\sigma(I)$][a]	0.0321	0.0217
wR_2[a]	0.0518	0.0574
GOF	1.016	1.041

[a] $R_1 = \Sigma||F_o|-|F_c||/\Sigma|F_o|$, $wR_2 = \{\Sigma[w(F_o^2-F_c^2)]/\Sigma[w(F_o^2)^2]\}^{1/2}$

Table S3. Bond valence sum (BVS) values of Si atoms and W atoms of $(TPP)_4[SiW_{12}O_{40}]$.

Si1A	3.83	Si1B	3.85				
W1A	6.10	W2A	6.22	W3A	6.17	W4A	6.12
W5A	6.13	W6A	6.12	W7A	6.12	W8A	6.22
W9A	6.21	W10A	6.14	W11A	6.20	W12A	6.19
W1B	6.12	W2B	6.21	W3B	6.18	W4B	6.11
W5B	6.12	W6B	6.13	W7B	6.12	W8B	6.22
W9B	6.21	W10B	6.12	W11B	6.20	W12B	6.21

Table S4. BVS values of Si atoms and W atoms of $(TPP)_4[SiW_{12}O_{28}Se_{12}]$.

Si1A	3.99	Si1B	4.04				
W1	6.37	W2	6.37	W3	6.31	W4	6.22
W5	6.26	W6	6.26	W7	6.24	W8	6.22
W9	6.30	W10	6.31	W11	6.34	W12	6.34
W21	6.22	W22	6.27	W23	6.32	W24	6.32
W25	6.22	W26	6.42	W27	6.23	W28	6.35
W29	6.29	W30	6.37	W31	6.38	W32	6.22

Table S5. BVS values of Se atoms of $(TPP)_4[SiW_{12}O_{28}Se_{12}]$.

Se1	1.98	Se2	1.93	Se3	1.90	Se4	1.89
Se5	1.85	Se6	1.88	Se7	1.86	Se8	1.82
Se9	1.90	Se10	1.92	Se11	1.95	Se12	1.86
Se21	1.69	Se22	1.85	Se23	1.87	Se24	1.77
Se25	2.00	Se26	1.89	Se27	1.72	Se28	1.95
Se29	1.95	Se30	1.79	Se31	1.80	Se32	1.62

Table S6. Average W=E bond length (Å) based on crystallographic analysis of $(TPP)_4[SiW_{12}O_{28}E_{12}]$ (E = O, S, and Se).

Compound	Average bond length (Å)			
	Si–μ_4-O	W=E	W–μ_4-O	W–μ_2-O
$(TPP)_4[SiW_{12}O_{40}]$	1.62	1.71	2.35	1.92
$(TPP)_4[SiW_{12}O_{28}S_{12}]$	1.64	2.15	2.36	1.91
$(TPP)_4[SiW_{12}O_{28}Se_{12}]$	1.62	2.28	2.36	1.91

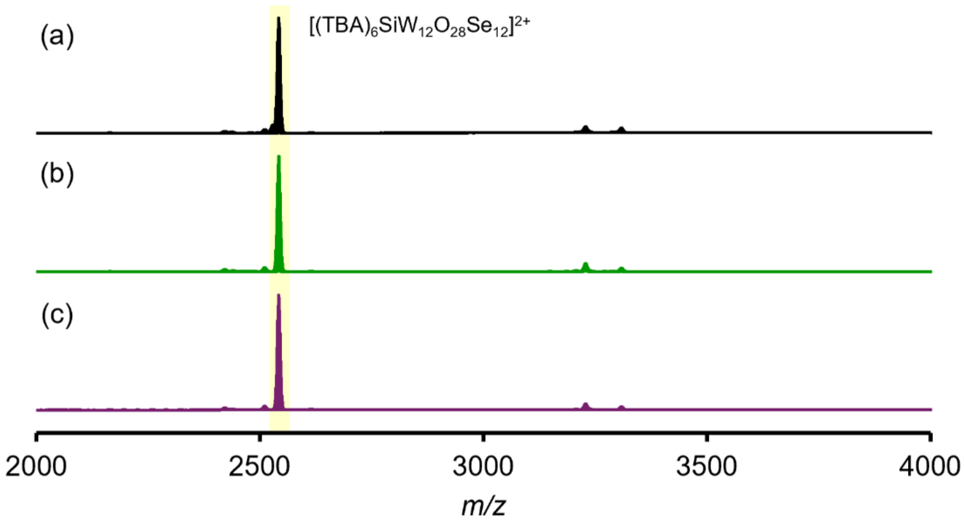

Fig. S1 (a-c) ESI-mass spectra of $(TBA)_4[SiW_{12}O_{28}Se_{12}]$ (a) in acetonitrile, (b) in acetonitrile after standing for a week, and (c) in acetonitrile solution containing 1 vol% H_2O (ca. 10000 equivalent respect to $(TBA)_4[SiW_{12}O_{28}Se_{12}]$) after standing for a week.

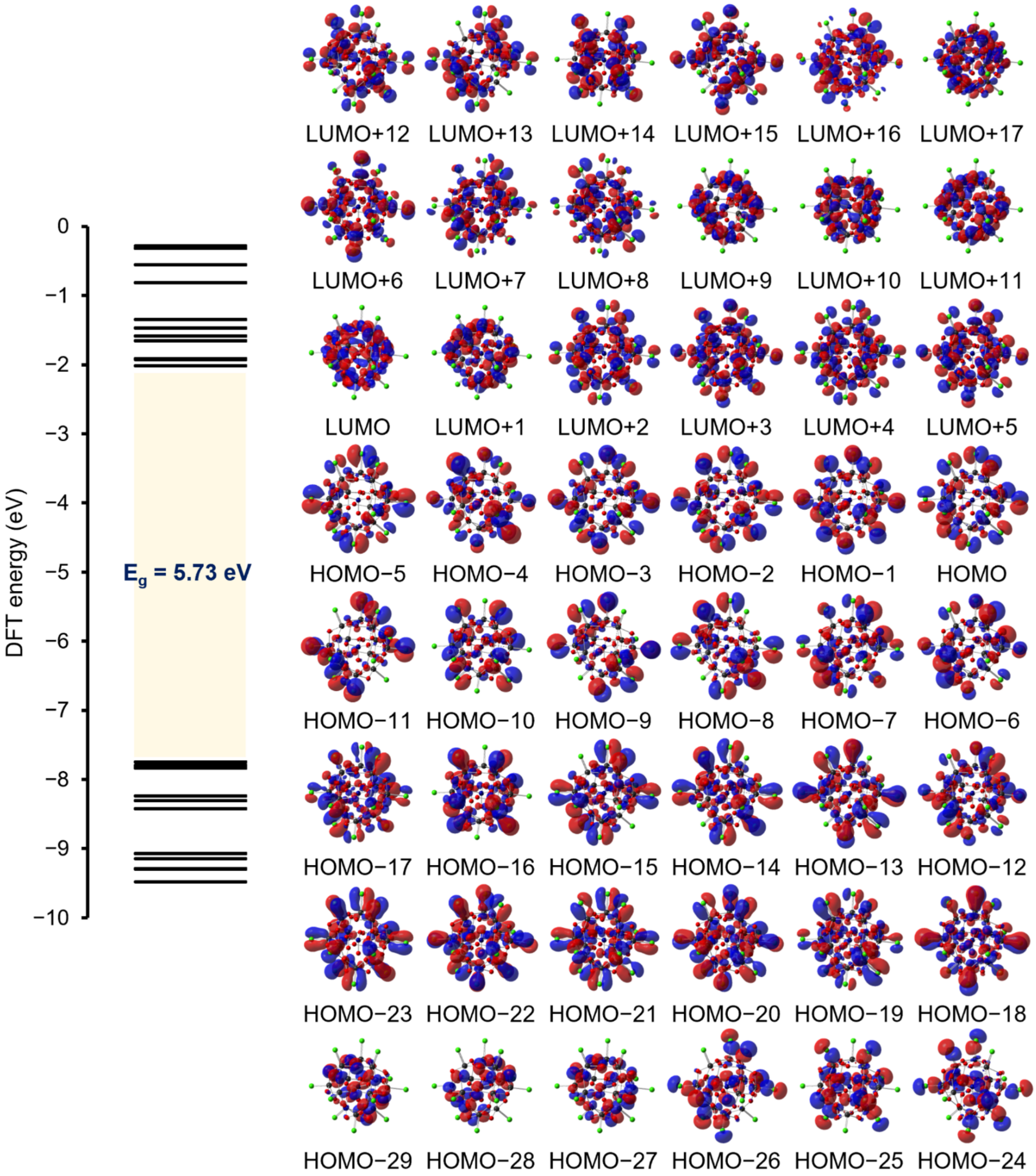

Fig. S2 Energy diagram and molecular orbitals of $[SiW_{12}O_{28}Se_{12}]^{4-}$ based on DFT study.

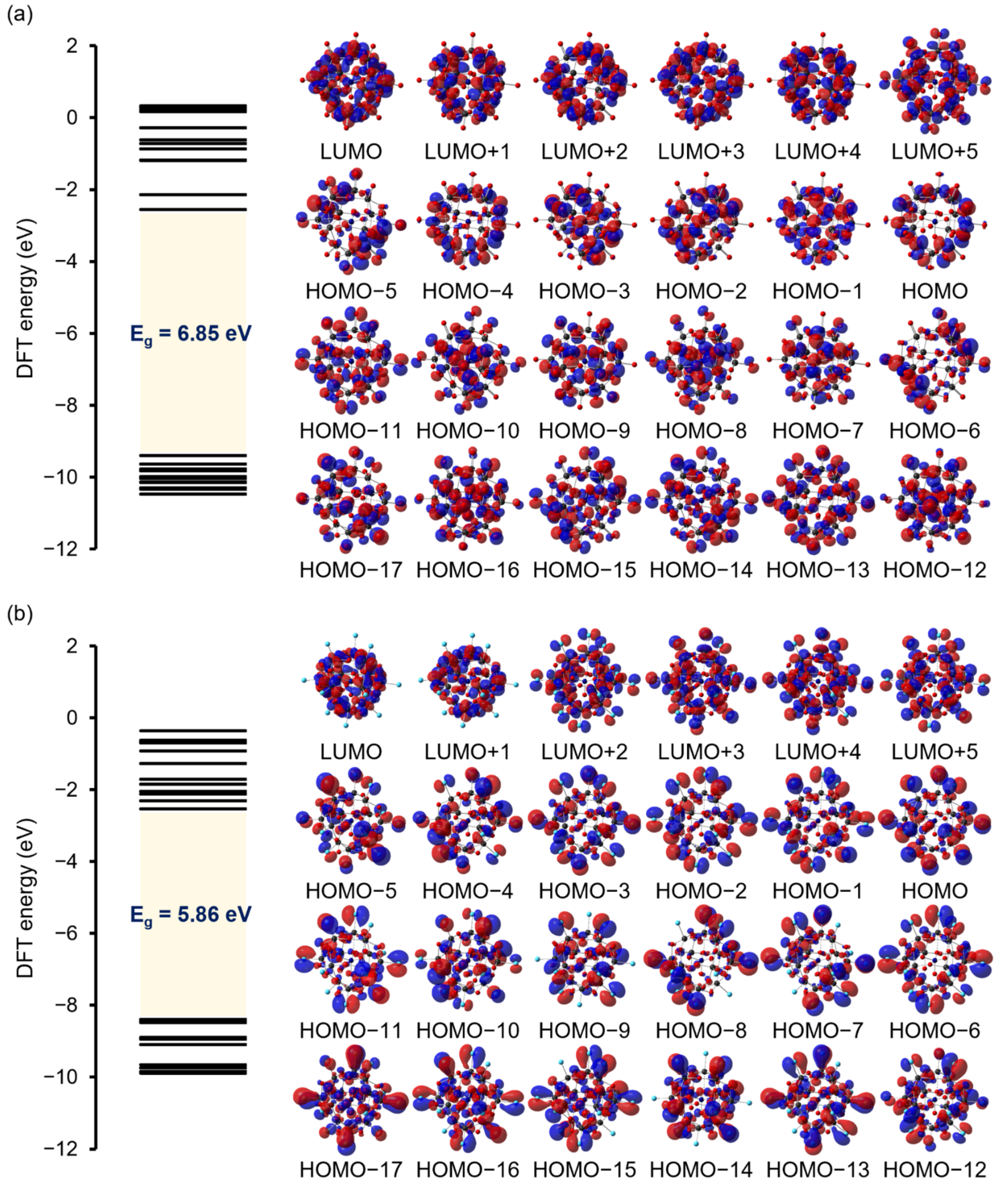

Fig. S3 Energy diagrams and molecular orbitals of (a) $[SiW_{12}O_{40}]^{4-}$ and (b) $[SiW_{12}O_{28}S_{12}]^{4-}$ based on DFT studies in our previous report.[S2]

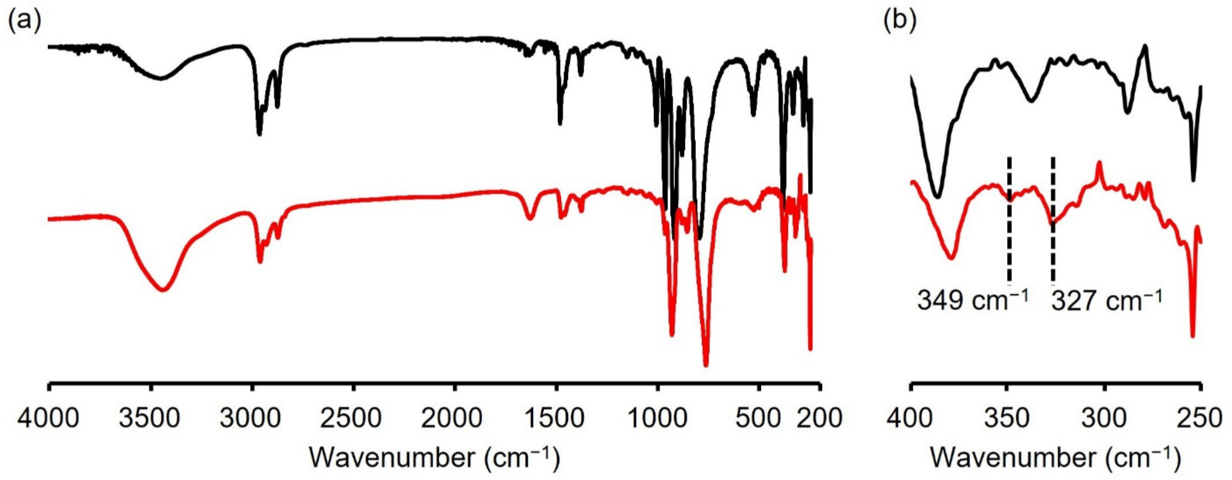

Fig. S4 FT-IR spectra of $(TBA)_4[SiW_{12}O_{40}]$ (black line) and $(TBA)_4[SiW_{12}O_{28}Se_{12}]$ (red line) in (a) a wide view and (b) an enlarged view.

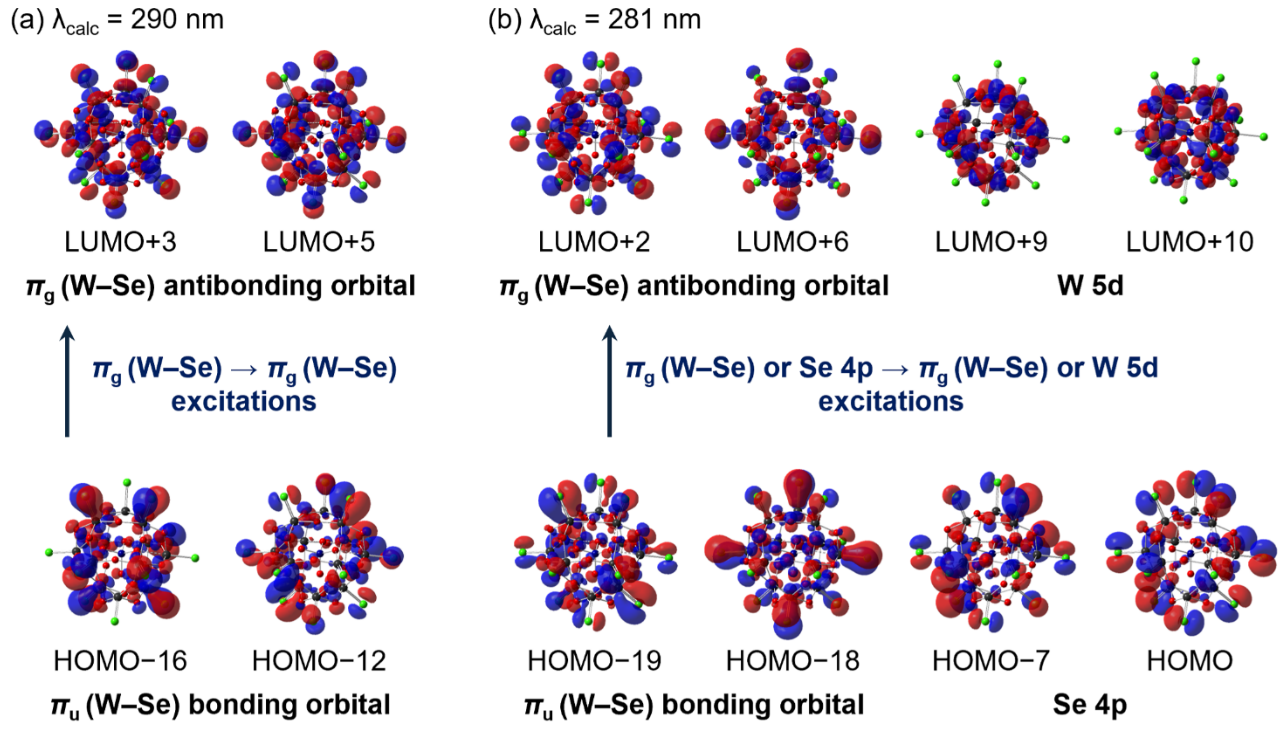

Fig. S5 Schematic representation of prominent excitations and selected related molecular orbitals based on TD-DFT study of $[SiW_{12}O_{28}Se_{12}]^{4-}$.

References

S1. C. Rocchiccioli-Deltcheff, M. Fournier, R. Franck and R. Thouvenot, *Inorg. Chem.*, 1983, **22**, 207.

S2. K. Yonesato, K. Yamaguchi and K. Suzuki, *Chem. Sci.* 2024, **15**, 11267.

S3. Rigaku OD. CrysAlis PRO. Rigaku Oxford Diffraction Ltd, Yarnton, England (2018).

S4. O. V. Dolomanov, L. J. Bourhis, R. J. Gildea, J. A. K. Howard and H. Puschmann, *J. Appl. Cryst.*, 2009, **42**, 339.

S5. L. J. Farrugia, *J. Appl. Cryst.*, 2012, **45**, 849.

S6. G. M. Sheldrick, *Acta Crystallogr. A*, 2015, **71**, 3.

S7. (a) G. M. Sheldrick, *Acta Crystallogr. A*, 2008, **64**, 112; (b) G. M. Sheldrick, *Acta Crystallogr. C*, 2015, **71**, 3.

S8. N. E. Brese and M. O'Keeffe, *Acta Crystallogr. B*, 1991, **47**, 192.

S9. G. te Velde, F. M. Bickelhaupt, E. J. Baerends, C. Fonseca Guerra, S. J. A. van Gisbergen, J. G. Snijders and T. Ziegler, *J. Comput. Chem.*, 2001, **22**, 931–967.

S10. C. Fonseca Guerra, J. G. Snijders, G. te Velde and E. J. Baerends, *Theor. Chem. Acc.*, 1998, **99**, 391–403.

S11. ADF2022, SCM, Theoretical chemistry, Vrije Universiteit, Amsterdam, The Netherlands, https://www.scm.com.

S12. J. P. Perdew, K. Burke and M. Ernzerhof, *Phys. Rev. Lett.*, 1996, **77**, 3865–3868.

S13. M. Swart, A. W. Ehlers and K. Lammertsma, *Mol. Phys.*, 2004, **102**, 2467–2474.

S14. E. V. Lenthe, E. J. Baerends and J. G. Snijders, *J. Chem. Phys.*, 1993, **99**, 4597–4610.

S15. E. V. Lenthe, E. J. Baerends and J. G. Snijders, *J. Chem. Phys.*, 1994, **101**, 9783–9792.

S16. E. V. Lenthe, A. Ehlers and E. J. Baerends, *J. Chem. Phys.*, 1999, **110**, 8943–8953.

S17. (a) L. Vilà-Nadal, J. P. Sarasa, A. Rodríguez-Fortea, J. Igual, L. P. Kazansky and J. M. Poblet, *Chem. –Asian J.*, 2010, **5**, 97–104; (b) D. Shiels, M. Pascual-Borras, P. G. Waddell, C. Wills, J.M. Poblet and R. J. Errington, *Chem. Commun.*, 2023, **59**, 7919–7922.

S18. A. Klamt, *J. Phys. Chem.*, 1995, **99**, 2224–2235.

S19. A. Klamt, V. Jonas, T. Bürger and J. C. W. Lohrenz, *J. Phys. Chem. A*, 1998, **102**, 5074–5085.

S20. R. Ditchfield, *Mol. Phys.*, 1974, **27**, 789–807.

S21. K. Wolinski, J. F. Hinton and P. Pulay, *J. Am. Chem. Soc.*, 1990, **112**, 8251–8260.

S22. G. Schreckenbach and T. Ziegler, *J. Phys. Chem.*, 1995, **99**, 606–611.
